# Redetermination of *cyclo*-trimethyl­ene­trinitramine

**DOI:** 10.1107/S1600536808019727

**Published:** 2008-07-09

**Authors:** Patrick Hakey, Wayne Ouellette, Jon Zubieta, Timothy Korter

**Affiliations:** a Department of Chemistry, Syracuse University, New York 13244, USA

## Abstract

The redetermined structure of 1,3,5-trinitro-1,3,5-triaza­cyclo­hexane, C_3_H_6_N_6_O_6_, at 90 (2) K has ortho­rhom­bic (*Pbca*) symmetry. It is of inter­est with respect to energetic compounds. The structure was originally investigated through X-ray diffraction by Hultgren [(1936). *J. Chem. Phys.* **4**, 84]. Later X-ray investigations were completed by McCrone [(1950). *Anal. Chem.* **22**, 954–955] and Harris, Reed & Gluyas [(1959). AFOSR-TR-59-165 Ohio State University Research Foundation, Columbus, Ohio, USA]. A single-crystal neutron diffraction study was performed by Choi & Prince [(1972). *Acta Cryst.* B**28**, 2857–2862] to ascertain the H-atom positions, which had not been defined by the earlier X-ray diffraction studies. All previous studies were performed at or near room temperature. The structure provided is the α polymorph of the title compound. The ring atoms are arranged in the chair conformation with two nitro groups occupying pseudo-equatorial positions and the remaining nitro group is axial. The crystal packing is stabilized by close intramolecular interactions from one H atom in each methylene group to O atoms of adjacent nitro groups, ranging from 2.251 to  2.593 Å.

## Related literature

For related literature, see: Akhavan (2004 ▶); Bachmann & Sheehan (1949 ▶); Brockman *et al.* (1949 ▶); Choi & Prince (1972 ▶); Ciezak *et al.* (2007 ▶); Davidson *et al.* (2008 ▶); Harris *et al.* (1959 ▶); Henning (1899 ▶); von Herz *et al.* (1920 ▶); Hultgren (1936 ▶); McCrone (1950 ▶); Yi & Cai (2008 ▶).
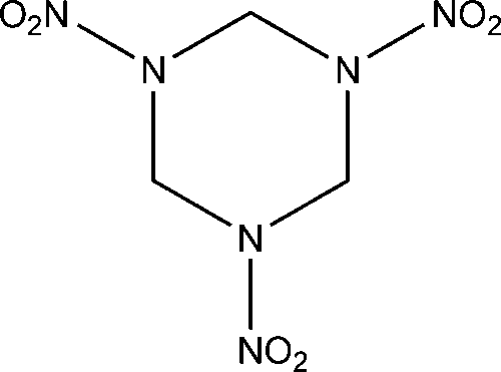


## Experimental

### 

#### Crystal data

C_3_H_6_N_6_O_6_
*M* *_r_* = 222.14
Orthorhombic, 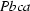

*a* = 11.4195 (8) Å
*b* = 10.5861 (7) Å
*c* = 13.1401 (9) Å
*V* = 1588.48 (19) Å^3^
*Z* = 8
Mo *K*α radiation
μ = 0.18 mm^−1^
*T* = 90 (2) K
0.34 × 0.20 × 0.20 mm

#### Data collection

Bruker APEX CCD area-detector diffractometer
Absorption correction: multi-scan (*SADABS*; Bruker, 2002 ▶)*T*_min_ = 0.943, *T*_max_ = 0.966
15555 measured reflections
1973 independent reflections
1783 reflections with *I* > 2σ(*I*)
*R*_int_ = 0.028

#### Refinement

*R*[*F* ^2^ > 2σ(*F* ^2^)] = 0.032
*wR*(*F* ^2^) = 0.082
*S* = 1.05
1973 reflections
160 parameters
All H-atom parameters refined
Δρ_max_ = 0.34 e Å^−3^
Δρ_min_ = −0.20 e Å^−3^

### 

Data collection: *SMART* (Bruker, 2002 ▶); cell refinement: *SAINT* (Bruker, 2002 ▶); data reduction: *SAINT*; program(s) used to solve structure: *SHELXS97* (Sheldrick, 2008 ▶); program(s) used to refine structure: *SHELXL97* (Sheldrick, 2008 ▶); molecular graphics: *CrystalMaker* (Palmer, 2006 ▶); software used to prepare material for publication: *SHELXTL* (Sheldrick, 2008 ▶).

## Supplementary Material

Crystal structure: contains datablocks I, global. DOI: 10.1107/S1600536808019727/lh2649sup1.cif

Structure factors: contains datablocks I. DOI: 10.1107/S1600536808019727/lh2649Isup2.hkl

Additional supplementary materials:  crystallographic information; 3D view; checkCIF report

## Figures and Tables

**Fig. 1.**
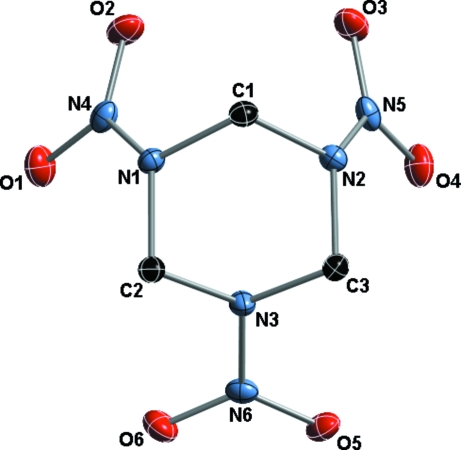
Perspective view of (I), with the atom numbering scheme and thermal ellipsoids drawn at 50% probability level. H atoms have been omitted for clarity.

## supplementary crystallographic information

### Comment

This single-crystal X-ray diffraction study of the explosive material 1,3,5-trinitro-1,3,5-triazacyclohexane presents a complete determination of the lattice dimensions and atomic coordinates at cryogenic temperatures. Specifically, the sample is maintained at 90 (2) K during the investigation. The compound is a highly explosive material and is known by many names, including cyclonite and RDX, among others (Bachmann & Sheehan, 1949). The formal name of the compound is hexahydro-1,3,5-trinitro-1,3,5-triazine. The structure provided in this study is the α polymorph of the title compound. Multiple polymorphs are possible for this compound; however, α is the form of the compound available at ambient conditions, while the β polymorph is quite unstable and its crystal structure is not known (McCrone, 1950). A third polymorph, γ is accessible at high pressures; its crystal structure has been ascertained through the collective use of both single-crystal X-ray and neutron powder diffraction studies (Davidson *et al.*, 2008). Indications that a fourth polymorph, δ, exists have been reported, but no structure is available (Ciezak *et al.*, 2007).

Several prior examinations of the crystal structure of the α polymorph of RDX have been performed using single-crystal X-ray diffraction (Hultgren, 1936; McCrone, 1950; Harris *et al.*, 1959). A slightly more recent study of RDX was performed using single-crystal neutron-diffraction (Choi & Prince, 1972). All prior studies on the title compound have been performed at or near room temperature, prompting the reexamination of this compound at low temperature to improve crystallographic precision. Through the determination of the crystallographic data at cryogenic temperature, insight into temperature induced lattice changes is gained, and precision of the atomic coordinates is increased. Improved precision is particularly useful for validation of first-principles solid-state modeling, while also helping to provide a more complete understanding of the molecular solid. Detailed knowledge of the solid-state crystal structure of this compound is imperative for its identification and detection *via* various spectroscopic methods, such as solid-state NMR, or terahertz. In addition, this study is intended to supplement the prior X-ray diffraction studies by supplying the atomic coordinates for all atoms in the structure, including the H atoms. Of the prior studies, only the neutron diffraction study provided the complete set of atomic coordinates.

RDX is a well known military explosive that has been in service for more than a half century. RDX is categorized as a secondary explosive; indicative of its stability relative to other explosive materials (Akhavan, 2004). The compound was first synthesized, and patented, by Henning (1899), and was originally developed for medicinal uses; consequently its capabilities as an explosive were not recognized until two decades later by von Herz *et al.* (1920). Since the original work, several additional methods have been reported for synthesis of the compound, including that by Bachmann & Sheehan (1949), Brockman *et al.* (1949), and most recently by Yi & Cai (2008). These newer methods are generally deemed to provide increased efficiency over the original syntheses.

In agreement with earlier studies, the structure was determined to retain the orthorhombic space group Pbca with *z* value 8; these parameters are in accordance with the prior work (Choi & Prince, 1972). The unit-cell dimensions determined in this study are as follows: a = 11.4195 (8), b = 10.5861 (7), and c = 13.1401 (9). These lattice values yield an overall cell volume of 1588.48 Å3. The cell volume calculated here is approximately 2.8% less than the most recently determined value for the title compound (Choi & Prince, 1972). The deviation in cell volume is likely attributable to contraction of the unit cell caused by the large temperature differential between the current cryogenic study and the earlier study, which was conducted at room temperature.

### Experimental

The material used in this work was purchased from AccuStandard, Inc. (99.4% purity by HPLC). The title compound, 1,3,5-trinitro-1,3,5-triazacyclohexane, was provided dissolved in a 1:1 solution of methanol and acetonitrile. Slow, room temperature evaporation of the solution was employed to permit the growth of the required crystals.

### Refinement

H atoms were located in a difference map and refined freely.

### Crystal data

**Table:**

C_3_H_6_N_6_O_6_	*F*_000_ = 912
*M**_r_* = 222.14	*D*_x_ = 1.858 Mg m^−^^3^
Orthorhombic, *P**b**c**a*	Mo *K*α radiation λ = 0.71073 Å
Hall symbol: -P2ac2ab	Cell parameters from 4268 reflections
*a* = 11.4195 (8) Å	θ = 3.1–28.2º
*b* = 10.5861 (7) Å	µ = 0.18 mm^−^^1^
*c* = 13.1401 (9) Å	*T* = 90 (2) K
*V* = 1588.48 (19) Å^3^	Block, colorless
*Z* = 8	0.34 × 0.20 × 0.20 mm

### Data collection

**Table:**

Bruker APEX CCD area-detector diffractometer	1973 independent reflections
Monochromator: graphite	1783 reflections with *I* > 2σ(*I*)
Detector resolution: 512 pixels mm^-1^	*R*_int_ = 0.028
*T* = 90(2) K	θ_max_ = 28.3º
φ and ω scans	θ_min_ = 3.1º
Absorption correction: multi-scan(SADABS; Bruker, 2002)	*h* = −15→15
*T*_min_ = 0.943, *T*_max_ = 0.966	*k* = −14→14
15555 measured reflections	*l* = −17→17

### Refinement

**Table:**

Refinement on *F*^2^	Secondary atom site location: difference Fourier map
Least-squares matrix: full	Hydrogen site location: inferred from neighbouring sites
*R*[*F*^2^ > 2σ(*F*^2^)] = 0.032	All H-atom parameters refined
*wR*(*F*^2^) = 0.082	*w* = 1/[σ^2^(*F*_o_^2^) + (0.0397*P*)^2^ + 0.7623*P*] where *P* = (*F*_o_^2^ + 2*F*_c_^2^)/3
*S* = 1.06	(Δ/σ)_max_ = 0.001
1973 reflections	Δρ_max_ = 0.34 e Å^−^^3^
160 parameters	Δρ_min_ = −0.19 e Å^−^^3^
Primary atom site location: structure-invariant direct methods	Extinction correction: none

### Special details

**Table:**

Geometry. All e.s.d.'s (except the e.s.d. in the dihedral angle between two l.s. planes) are estimated using the full covariance matrix. The cell e.s.d.'s are taken into account individually in the estimation of e.s.d.'s in distances, angles and torsion angles; correlations between e.s.d.'s in cell parameters are only used when they are defined by crystal symmetry. An approximate (isotropic) treatment of cell e.s.d.'s is used for estimating e.s.d.'s involving l.s. planes.
Refinement. Refinement of *F*^2^ against ALL reflections. The weighted *R*-factor *wR* and goodness of fit *S* are based on *F*^2^, conventional *R*-factors *R* are based on *F*, with *F* set to zero for negative *F*^2^. The threshold expression of *F*^2^ > σ(*F*^2^) is used only for calculating *R*-factors(gt) *etc*. and is not relevant to the choice of reflections for refinement. *R*-factors based on *F*^2^ are statistically about twice as large as those based on *F*, and *R*- factors based on ALL data will be even larger.

### Fractional atomic coordinates and isotropic or equivalent isotropic displacement parameters (Å^2^)

**Table:**

	*x*	*y*	*z*	*U*_iso_*/*U*_eq_	
O1	0.96483 (8)	0.36616 (9)	1.02694 (7)	0.0224 (2)	
O2	0.85097 (8)	0.24565 (9)	1.11679 (6)	0.0206 (2)	
O3	0.81846 (8)	−0.03257 (8)	1.07225 (7)	0.0208 (2)	
O4	0.93294 (8)	−0.10398 (8)	0.95344 (7)	0.0213 (2)	
O5	1.06779 (7)	0.06532 (8)	0.73282 (6)	0.01755 (19)	
O6	1.09321 (8)	0.26371 (8)	0.77072 (7)	0.0205 (2)	
N1	0.81806 (8)	0.26489 (9)	0.94911 (7)	0.0121 (2)	
N2	0.79657 (8)	0.04040 (9)	0.91362 (7)	0.0127 (2)	
N3	0.93467 (8)	0.16430 (9)	0.82559 (7)	0.0134 (2)	
N4	0.88448 (9)	0.29180 (9)	1.03687 (7)	0.0144 (2)	
N5	0.85527 (9)	−0.03513 (9)	0.98530 (8)	0.0151 (2)	
N6	1.03699 (9)	0.16474 (10)	0.77253 (7)	0.0138 (2)	
C1	0.74033 (10)	0.15500 (11)	0.95281 (8)	0.0126 (2)	
C2	0.87808 (10)	0.28363 (10)	0.85261 (9)	0.0135 (2)	
C3	0.85587 (10)	0.05633 (11)	0.81621 (9)	0.0140 (2)	
H1A	0.6771 (13)	0.1709 (13)	0.9097 (11)	0.012 (3)*	
H1B	0.7145 (13)	0.1409 (13)	1.0185 (11)	0.012 (3)*	
H2A	0.8192 (12)	0.3030 (13)	0.8023 (11)	0.010 (3)*	
H2B	0.9329 (13)	0.3488 (14)	0.8585 (11)	0.016 (4)*	
H3A	0.7972 (13)	0.0760 (14)	0.7669 (12)	0.020 (4)*	
H3B	0.8988 (13)	−0.0155 (14)	0.8005 (11)	0.014 (3)*	

### Atomic displacement parameters (Å^2^)

**Table:**

	*U*^11^	*U*^22^	*U*^33^	*U*^12^	*U*^13^	*U*^23^
O1	0.0196 (4)	0.0205 (5)	0.0271 (5)	−0.0051 (4)	−0.0064 (4)	−0.0029 (4)
O2	0.0257 (5)	0.0252 (5)	0.0110 (4)	0.0027 (4)	−0.0017 (3)	−0.0004 (3)
O3	0.0261 (5)	0.0210 (4)	0.0152 (4)	−0.0046 (4)	−0.0036 (4)	0.0046 (3)
O4	0.0172 (4)	0.0146 (4)	0.0320 (5)	0.0030 (3)	−0.0054 (4)	−0.0011 (4)
O5	0.0170 (4)	0.0202 (4)	0.0154 (4)	0.0052 (3)	0.0031 (3)	−0.0015 (3)
O6	0.0166 (4)	0.0196 (4)	0.0255 (5)	−0.0036 (3)	0.0044 (4)	0.0044 (3)
N1	0.0128 (4)	0.0137 (4)	0.0099 (4)	0.0002 (4)	−0.0010 (3)	−0.0014 (3)
N2	0.0120 (4)	0.0129 (4)	0.0133 (5)	0.0004 (3)	−0.0002 (3)	0.0017 (3)
N3	0.0128 (5)	0.0122 (4)	0.0152 (4)	0.0001 (3)	0.0041 (4)	−0.0003 (4)
N4	0.0148 (5)	0.0140 (5)	0.0143 (5)	0.0040 (4)	−0.0031 (4)	−0.0030 (4)
N5	0.0140 (5)	0.0116 (4)	0.0197 (5)	−0.0033 (4)	−0.0050 (4)	0.0019 (4)
N6	0.0125 (4)	0.0181 (5)	0.0109 (4)	0.0011 (4)	0.0003 (3)	0.0033 (4)
C1	0.0101 (5)	0.0152 (5)	0.0126 (5)	0.0005 (4)	0.0010 (4)	−0.0002 (4)
C2	0.0158 (5)	0.0114 (5)	0.0132 (5)	0.0018 (4)	0.0022 (4)	0.0008 (4)
C3	0.0151 (5)	0.0131 (5)	0.0139 (5)	−0.0010 (4)	0.0021 (4)	−0.0019 (4)

### Geometric parameters (Å, °)

**Table:**

O1—N4	1.2159 (13)	N2—C1	1.4660 (14)
O2—N4	1.2199 (13)	N3—N6	1.3606 (13)
O3—N5	1.2177 (14)	N3—C3	1.4600 (14)
O4—N5	1.2219 (14)	N3—C2	1.4626 (14)
O5—N6	1.2263 (13)	C1—H1A	0.934 (15)
O6—N6	1.2290 (13)	C1—H1B	0.925 (14)
N1—N4	1.4093 (13)	C2—H2A	0.964 (15)
N1—C2	1.4551 (14)	C2—H2B	0.934 (15)
N1—C1	1.4641 (14)	C3—H3A	0.956 (16)
N2—N5	1.4056 (13)	C3—H3B	0.928 (15)
N2—C3	1.4579 (14)		
			
N4—N1—C2	115.59 (9)	N1—C1—N2	112.35 (9)
N4—N1—C1	117.37 (9)	N1—C1—H1A	107.8 (9)
C2—N1—C1	115.01 (9)	N2—C1—H1A	105.9 (9)
N5—N2—C3	115.63 (9)	N1—C1—H1B	110.7 (9)
N5—N2—C1	116.38 (9)	N2—C1—H1B	109.5 (9)
C3—N2—C1	114.59 (9)	H1A—C1—H1B	110.4 (13)
N6—N3—C3	119.26 (9)	N1—C2—N3	107.57 (9)
N6—N3—C2	120.05 (9)	N1—C2—H2A	107.3 (8)
C3—N3—C2	115.10 (9)	N3—C2—H2A	109.0 (8)
O1—N4—O2	126.04 (10)	N1—C2—H2B	110.1 (9)
O1—N4—N1	116.69 (10)	N3—C2—H2B	111.2 (9)
O2—N4—N1	117.04 (10)	H2A—C2—H2B	111.5 (12)
O3—N5—O4	125.82 (10)	N2—C3—N3	107.62 (9)
O3—N5—N2	116.84 (10)	N2—C3—H3A	107.2 (9)
O4—N5—N2	117.12 (10)	N3—C3—H3A	108.6 (9)
O5—N6—O6	125.00 (10)	N2—C3—H3B	110.2 (9)
O5—N6—N3	117.48 (10)	N3—C3—H3B	109.5 (9)
O6—N6—N3	117.48 (10)	H3A—C3—H3B	113.5 (13)
			
C2—N1—N4—O1	24.84 (13)	N4—N1—C1—N2	−93.07 (11)
C1—N1—N4—O1	165.72 (10)	C2—N1—C1—N2	48.04 (12)
C2—N1—N4—O2	−160.30 (10)	N5—N2—C1—N1	91.11 (11)
C1—N1—N4—O2	−19.42 (14)	C3—N2—C1—N1	−48.17 (12)
C3—N2—N5—O3	167.78 (10)	N4—N1—C2—N3	90.33 (11)
C1—N2—N5—O3	28.92 (13)	C1—N1—C2—N3	−51.49 (12)
C3—N2—N5—O4	−17.30 (14)	N6—N3—C2—N1	−148.08 (10)
C1—N2—N5—O4	−156.16 (10)	C3—N3—C2—N1	58.46 (12)
C3—N3—N6—O5	−12.18 (14)	N5—N2—C3—N3	−87.58 (11)
C2—N3—N6—O5	−164.56 (10)	C1—N2—C3—N3	52.01 (12)
C3—N3—N6—O6	170.00 (10)	N6—N3—C3—N2	147.49 (9)
C2—N3—N6—O6	17.63 (15)	C2—N3—C3—N2	−58.82 (12)
